# Reduced PARP1 as a Serum Biomarker for Graft Rejection in Kidney Transplantation

**DOI:** 10.4172/jpb.1000350

**Published:** 2015

**Authors:** Meera Srivastava, Yelizaveta Torosyan, Ofer Eidelman, Catherine Jozwik, Harvey B. Pollard, Rosyln Mannon

**Affiliations:** 1 Department of Anatomy, Physiology and Genetics, and Institute for Molecular Medicine, Uniformed Services University School of Medicine (USUHS), Bethesda, MD, USA; 2 Division of Nephrology, University of Alabama at Birmingham, Birmingham, AL 35294, USA

**Keywords:** Kidney transplantation, Graft rejection/injury, Serum biomarkers, Proteomics, Gene Pattern, Ingenuity pathway analysis

## Abstract

A serum proteomics platform enabling expression Profiling in transplantation-associated clinical subsets gives an opportunity to identify non-invasive biomarkers that can accurately predict transplant outcome. In this study, we attempted to identify candidate serum biomarkers that could predict kidney allograft rejection/injury, regardless of its etiological and therapeutic heterogeneity. Using serum samples collected from kidney transplantation patients and healthy controls, we first employed Clontech-500 Ab microarrays to Profile acute rejection (AR) and chronic graft injury (CGI) versus stable graft function (SF) and normal kidneys (NK). Using GenePattern analysis of duplicate arrays on pooled samples, we identified gender-independent biomarkers PARP1, MAPK1, SRP54, DP1, and p57 (FDR ≈ 25%), the concordant downregulation of which represented a detrimental Profile common for both rejection/ injury types (AR-CGI). The reverse phase arrays qualified a 2-fold upregulation of PARP1 with an ROC of 0.87 in individual samples from patients with SF vs. AR-CGI rendering serum PARP1 as a biomarker for early prognosis. Ingenuity Pathways Analysis (IPA) connected PARP1 to some other markers (MAPK1), elucidating their possible interactions and connections to the immune response and graft-versus-host disease signaling. The downregulation of serum PARP1 in the damaged graft tissues, represents a perspective non-invasive marker, predicting the failing kidney graft, regardless of rejection/injury causes or gender. Thus, the successful identification of PARP1 as a bio-marker in limited patient cohorts demonstrates that serum proteomics platform empowered by the GenePattern- and IPA-based Bioinformatics algorithm can guarantee a successful development of the clinically applicable prognostic biomarker panel.

## Introduction

The fast growing scientific field of proteomics can provide novel avenues in the transplant-related immunomodulation and novel “beyond histology” tools to diagnose allograft dysfunction [1]. Clearly identifiable and biologically meaningful protein expression patterns can lead to a better understanding of molecular mechanisms responsible for etiological heterogeneity in kidney allograft rejection. According to the Banff '09 meeting report on allograft pathology [2], molecular research data compels the incorporation of ‘omics-technologies and discovery of novel markers with the goal of combining histopathology and molecular parameters within the working classification. Serum proteomics in particular is an attractive non-invasive monitoring tool that can identify markers reflecting the failing kidney graft pathophysiology as well as the host-allograft interactions and inadequate immune response, which can be used to prevent the graft-associated complications [3,4].

A number of immune response assays was developed to monitor and to predict the graft-associated complications [3,4]. However, many of the currently used assays for early prognosis fail in reproducibility and are not formally validated in large transplanted cohorts. A more useful approach to treating graft rejection would appear to be early, non-invasive identification of graft rejection. The recent progress of proteomics has opened up novel avenues for rejection-related biomarker discovery However, adopting high-throughput proteomic approaches to multiplexed set-ups, providing a minimally invasive screening procedure, targeting non-fractionated biological fluids, has proven to be challenging. Antibody-based microarrays are a rapidly emerging affnity-proteomic technology that is likely to play an increasing role in proteomics. In recent years, the technology has made significant progress [5] now allowing us to design miniaturised array platforms, capable of simultaneously Profiling numerous low-abundant protein analytes in complex proteomes, such as plasma and serum, while consuming only minute amounts of sample. Adopting antibody microarrays, translational proteomics is one immediate application where comparative protein expression Profiling analysis of disease versus normal proteomes could yield tentative predictive biomarker signatures. From a clinical point of view, we also need increased possibilities to individually monitor disease progression and response to treatment, since no therapy has the same effect on a large number of patients with the same diagnosis.

In this pilot study, we intended to identify candidate serum biomarkers that could predict clinically defined types of graft failure (acute rejection, AR, and chronic graft injury, CGI) regardless of their etiological heterogeneity and treatment modalities. We were able to identify gender-independent biomarkers, common for AR-CGI using Clontech-500 Antibody arrays and data mining by GenePattern and Ingenuity Pathway Analysis (IPA). Differential expression of PARP, in particular, was first identified by Clontech-500 Ab arrays in pooled samples, and then was confirmed by in-house reverse protein capture arrays in individual samples. As a result, serum PARP1 down-regulation was validated as a marker for rejection/injury (AR-CGI) versus stable function (SF) and normal (NK).

## Material and Methods

### Patient details

The serum samples were collected from healthy individuals with normal kidney function (NK) and kidney transplant patients with stable function (SF) or clinically defined acute rejection and chronic graft injury (AR and CGI, respectively). All transplant recipients received multiple immuno-suppressive treatments. These included non depletional(anti-CD25 antibody) and depletional induction with monoclonal or polyclonal antibodies (Alemtuzumab or Thymoglobulin) followed by tacrolimus and/or mycophenolate mofetil or sirolimus typically in a steroid free strategy. Forty individuals were included in the study: The patients were divided in four groups: (1) Eight healthy donors with no medical problems, without any medications, and with normal renal function constituted the controls (NK). (2) 11 patients with at least 6 months post-transplant without change in renal function and the absence of any significant histological or clinical abnormalities constituted stable function (SF). (3) 11 patients with a rise in serum creatinine of at least 18% from baseline, with characteristic histologic changes showing at least Banff grade 2 chronic glomerulopathy, and at least grade I interstitial fibrosis and tubular atrophy [6] constituted chronic graft injury (ChR). Acute rejection (AR, 10 patients) was defined based on renal biopsies that histologically satisfied the Banff criteria (Borderline, IA, IB, IIA or IIB) [6]. All patients were enrolled in Institutional Review Board approved clinical trials at the National Institutes of Health after informed concent prior to being investigated in this study.

## Protein Profiling using Antibody Microarrays

### Labeling of serum proteins

**Figure 1:**
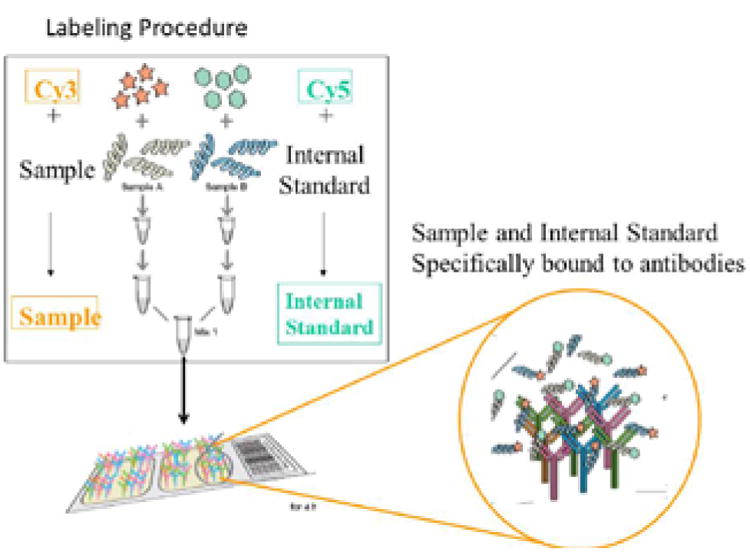
Methodology for serum labelling procedure from kidney transplant patient differing in graft survival vs. normal controls. Proteins from kidney transplant serum at different stages of the disease (sample) vs. normal serum (control) were labeled with Cy3 or Cy5 dyes, respectively, and analyzed on an antibody microarray platform.

Serum of each of patients in each annotated set were pooled and labeled with Cy3 in equal volumes. Samples of FDA-certified, pooled normal control male serum was labeled with Cy5. Equal volumes of pooled Cy3-labeled patient sera were mixed with Cy5-labeled control serum. Samples of Cy3 control and Cy5 normal control sera were also mixed and assayed on the antibody microarray to permit correction for differences in labeling efficiency of Cy3 vs.Cy5. Each sample was incubated with a 507 feature antibody microarray (Clontech, Mountain View, CA), in a medium containing a detergent-based reagent to minimize protein-protein interactions. The methods were as described in our recent paper [7] and as schematically shown in Figure 1 (Clontech).

### Fluorescence detection

The fluorescence at each site on the antibody microarray was measured on a GenePix array reader (New Milton, New Hampshire, U.K.), and downloaded to an EXCEL spread sheet. The protein expression for each site on the antibody microarray was then ratio'ed to the same protein in normal control sera.

### Data analysis

Since each of the 507 microarray sites contains duplicate antibody spots, data from 4 independent technical replicates of each pool of patients were available for averaging and calculation of statistical significance. Sample selection therefore begins by rejection from calculations of all spots with intensities below the local background, as well as all spots with Signal-to-Noise-Ratio <3. We then calculated the standard deviation (SD) for each protein. Outliers were rejected if their deviations are larger than 2SD's from the average of the respective protein. The averages were recalculated by omitting outliers. If a given protein is still too noisy, the specific protein was excluded from the analysis. We quantitated volume-normalized protein levels by ratio'ing Cy3-labeled proteins in patient serum samples to the same protein, labeled with Cy5, in the normal control sera. Normal sera labeled with both Cy3 and Cy5 were ratio'ed to one another to calculate a labeling efficiency difference specific to each protein.

### Statistical methods

Two approaches were used. In the ***first*** approach, the differences between disease samples and normal controls were determined based on t-tests of the samples in each group. Statistical significance is defined as p<0.05, or P<0.01 for correlation analysis. P-values in the Tables are calculated from 2-tailed t-tests. In the ***second*** approach, which we presently prefer, we applied the SAM (Statistical Analysis of Microarrays: www.stat.stanford.edu/∼tibs/SAM) package to determine the false Discovery Rate. An FDR <10% was taken to be statistically significant.

## Qualification using Reverse Capture Protein Microarray

**Figure 2:**
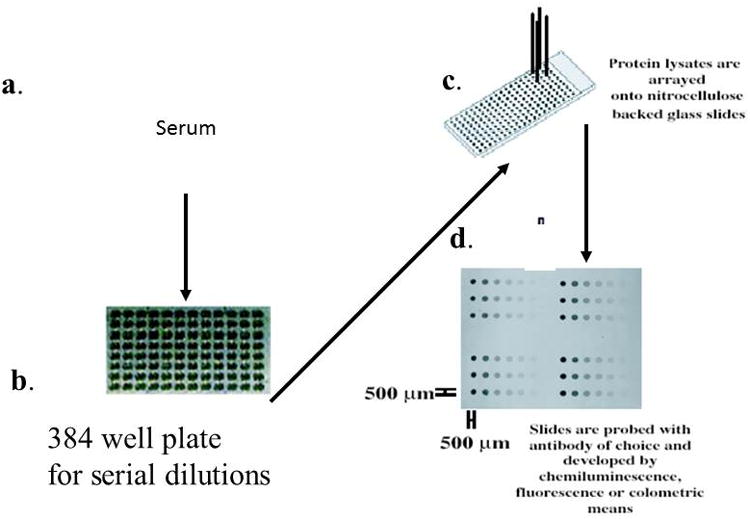
Methodology for Reverse Capture Protein Microarray analysis of stage-specific prostate cancer serum samples. (a) serum sample (b) Samples of individual sera from different stages of the disease were diluted as eight serial two-fold dilutions in a 384 well plate (c) samples printed on glass-backed, nitrocellulose (N/C) slides (d) slide image stained for reactivity with monoclonal antibody against PARP and developed by chemiluminescence.

Briefly, serum from each individual patient sample was printed in serially diluted fashion on slides [7-9]. Multiple patient serum samples were printed on single slides, and the entire dataset thereby probed with a given antibody. Antibodies for testing on this platform were chosen from those identified by the antibody microarray platform. Clontech (BD, Biosciences/Transduction Labs) supplies the exact same antibodies in soluble form as are printed on the microarrays. Antibody reactivity extinguishes at a given dilution, thus permitting estimation of a quatitative titer. Sample preparation consisted of mixing 30 μL sample of serum 1:1 with 2× SDS gel electrophoresis buffer and incubating for 10 minutes at 37°C. Serial 2-fold dilutions in 1× buffer were arrayed with an AUSHON printer (Waltham, MA) in serially diluted fashion (Janus Liquid Handling Workstation,) on a slide in hexaplicate. Patient serum samples were printed on multiple single slides, and the entire dataset was probed with PARP1 as shown in Figure 2. For detection of total protein on each spot, parallel arrays were stained with SYPRO RUBY protein blot stain (Molecular Probes). Controls were (i) buffer only; (ii) a dilution series with purified bovine serum albumin (BSA); (iii) a dilution series with normal pooled human serum.

### Total levels of antigens

Te total level of a given antigen in the serum was calculated by extrapolating the log of the measured intensities of the dilution series back to the y-axis (i.e., no dilution). The theoretical curve is linear with a slope of -1, with deviations occurring at the high end (due to saturation) and at the low end (due to noise). A slope of -1 indicates that there is a 1:1 relationship between printed antigen and bound antibody. Outliers and low signal to noise spots were excluded from the curve fitting.

### Ingenuity pathways analysis

To discriminate the molecular pathways responsible for stable function effects versus graft rejection, we used IPA software (www.ingenuity.com, Ingenuity Systems, Redwood City, CA). An average expression ratio of R>2 in stable function versus graft rejection comparisons was used as a threshold. The reports with outlier proteins from antibody microarray analysis were uploaded and mapped to corresponding objects (genes/proteins) in IPA's database and carried out GenePattern (the Broad Institute at MIT and Harvard) analysis.

## Results

In order to Profile serum protein expression in patients with stable function (SF) versus acute rejection or chronic graft injury (AR and CGI, respectively), we used Comparative Marker Selection (GenePattern) on Clontech Ab microarray derived datasets. Simultaneously, we used IPA to characterize putative biomarkers and to guide their selection by investigating their connections to the kidney-specific and graft-related signaling. Despite limited numbers in each patient category, we were able to identify SF, AR and CGI markers that showed plausible connections to the graft-related physiology and appeared to reflect rejection type-specific alterations as shown below. Moreover, some of the identified markers had similar Profiles in both AR and CGI categories, suggesting that they can be used as common markers for graft rejection/injury, regardless of etiological causes.

### Candidate serum biomarkers for Stable Function (SF) in kidney transplant patients

A panel of microarray-derived peripheral blood biomarkers was previously associated with spontaneous operational tolerance in patients with stable renal graft function in the absence of immunosuppression [10]. In our study using the Clontech Ab arrays and Gene Pattern analysis, the SF-category (Table 1) was characterized by upregulation of Fas-associated FADD, cytochrome B-245-beta CYBB, phosducin-like PDCL, and neurogenin NEUROG1, and by downregulation of linker for activation of T-cells LAT, lymphocyte-specific protein LSP1, lysosomal-associated membrane protein LAMP1, synaptonemal component SYCP3, tyrosine phosphatase PTPRZ1, and integrin-beta3 ITGB3 (or CD61). Some of these markers had connections to kidney damage. For instance, the major regulator of the T-cell antigen receptor (TCR) signal transduction LAT was upregulated in chronic graft injury [11]. LAMP1/CD107 is a degranulation marker for NK and activated CD8+ which can enhance antigen presentation to CD8+/CD4+ T-cells [12], and the IL2-stimulated LAMP1 paralleled the increase of cytotoxicity [13]. The Ptprz1 overexpression due to oxidative stress activated beta-catenin in renal tubular damage model [14]. Thus, the SF-signature prognosticating a favorable outcome included downregulation of several proteins, the upregulation of which is implicated in the T-cell mediated cytotoxicity and kidney damage.

### Candidate serum biomarkers for Acute Rejection (AR) in kidney transplant patients

The AR-category derived from the Clontech Ab arrays and Gene Pattern analysis (Table 2) was particularly characterized by the downregulation of stathmin STMN1 and Stmn1 deficiency was shown to increase renal failure and delayed recovery from ischemia-reperfusion injury in homozygous knockout *Stmn1* mice [15]. Human STMN1 was identified as a marker of tubular regeneration in the recovery phase of acute ischemic renal failure [16]. Additionally, the AR-signature in our study included downregulation of the critical activator of MAPK-cascade RAF1, which functions downstream of the Ras-family of GTPases, as well as the Ras GTPase activating RAP1GAP, and CSE1L (exportin-2), which is involved in the Ras-family member Ran signaling. On the other hand, the actin cytoskeleton regulator WASF1, which acts downstream of Rac, a Rho-family small GTPase, was upregulated, and the Rac1 GTPase activating NEK kinase was downregulated. In addition, downregulated AR-markers included DNAse NME1, the translin-binding nuclear regulator TSNAX, the SMAD-binding TGFB-regulator ZNF423, and proteasome PSMC5. Thus, the AR-signature expediently included major markers of acute renal failure (STMN1) as well as demonstrated alterations (NME1, Ras-Raf), which have been previously associated with the kidney transplantation specific treatments.

### Candidate serum biomarkers for Chronic Graft Injury (CGI) in kidney transplant patients

The CGI-signature derived from the Clontech Ab arrays and Gene Pattern analysis was characterized by downregulation of the immune response regulators NFATC1 and IKBKB, the cell death/proliferation associated PARP1, MCL1, TFDP1 and RBBP4, kinases MAPK1 and MATK, syntaxin-binding tomosyn STXBP5, and gephyrin GPHN (Table 3). These CGI-markers displayed close relations to the immune response and cell survival regulation that substantiated their putative biomarker roles.

### Common candidate AR-CGI biomarkers in kidney transplant patients

Next, we identified candidate gender-independent AR-CGI markers, which are expected to distinguish between the stable function and kidney graft failure, regardless of the rejection/injury type (Table 4). The FDRs reaching 25% increased the plausibility of top candidate AR-CGI-markers, some of which were previously identified as CGI-markers: PARP1, NFATC1, MAPK1, MATK, and TFDP1. In addition, the AR-CGI-signature included the cell proliferation regulating cyclin CCNE1, cyclin-dependent kinase inhibitor CDKN1C, oncogene ABL1 as well as ABL1-binding DLGAP1 and ribonucleoprotein SRP54.

### Serum PARP1 as a top marker in pooled AR-CGI categories

**Figure 3:**
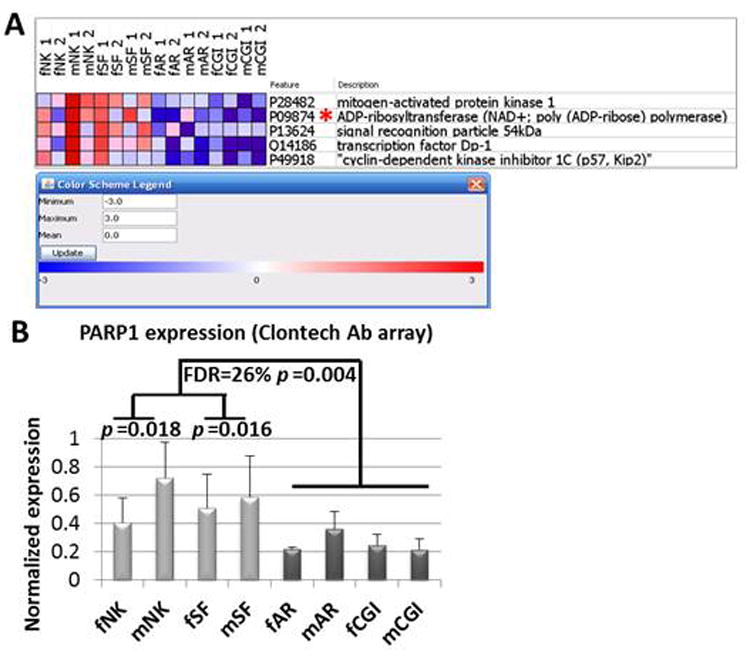
Serum PARP1 as a top marker in pooled AR-CGI categories (Clontech-500 Ab arrays, GenePattern) Microarray analysis using duplicate protein Ab arrays (Clontech-500) with pooled serum samples from kidney transplantation patients and controls was performed, as described in Material & Methods. Panel A: The heatmap shows top five markers in AR-CGI vs. Rest comparison (3-classes: NK, SF, AR-CGI) using ComparativeMarkerSelection (GenePattern). PARP1 is marked by an asterisk. In each category abbreviation: small letters ‘f’ and ‘m’ represent female and male genders, respectively; NK–normal kidney, SF–stable function, AR–acute rejection, and CGI–chronic graft injury. The legend shows color scheme for the heatmap (red–upregulated, blue–downregulated, and white–no change). Panel B: Normalized PARP1 expression from duplicate arrays in each category is presented as means (columns) with standard deviations (vertical bars). The f/mAR-CGI vs. f/mNK-SF comparison is characterized by FDR=26% and p=0.004, and f/mAR-CGI vs. f/mNK or f/mSF–by p=0.018 and .016, respectively.

Among the top-ranking five markers with most consistent downregulation Profiles in AR-CGI (Figure 3A), PARP1 (Figure 3B) demonstrated a >2-fold difference between AR-CGI and control NK and SF categories (p<0.018 and .016, respectively). Subsequently, the major cell survival indicator PARP1, known for its aforementioned relations to other markers such as MAPK1 and NFATC1, was considered a best candidate AR-CGI-marker and was chosen for qualification by reverse phase protein arrays in individual samples.

### Reverse Capture Protein Array approach for the validation of PARP1 biomarker role in early prognosis of kidney transplant rejection

**Figure 4:**
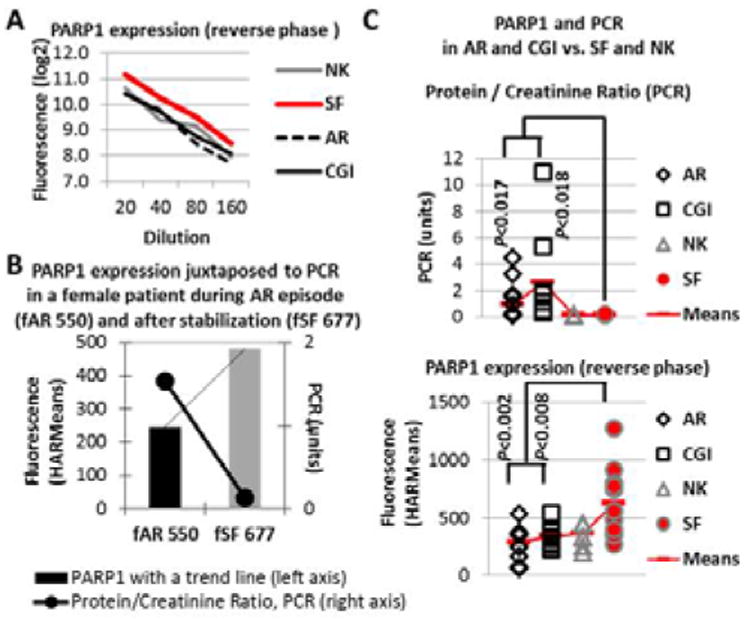
Validation of the identified AR-CGI-marker PARP1 in individual samples from kidney transplant patients (reverse capture protein arrays) In-house made Reverse Capture protein arrays with serially diluted individual serum samples were exposed to the PARP1 Ab previously used on Clontech arrays, as described in Material & Methods. The PARP1 values were assessed using HARMeans for fuorescence-based signal minus local background intensities. Panel A represents log2-transformed intensities (Y-axis) for each category (NK, SF, AR, and CGI) plotted against sample dilutions (20-40-80-160, X-axis). Panel B represents serum PARP1 expression values (columns with a trendline, left axis) with corresponding urine Protein / Creatinine Ratio, PCR values (black line, right axis) in the same female patient during AR-episode (fAR 550) and after kidney function stabilization (fSF 677). Panel C represents individual PARP1 HARMean values (bottom) and PCRs (top) with corresponding means in each gender-independent category (NK, SF, AR, CGI). Statistical significance of PCRs and PARP1 was assessed by T-tests (two-tailed distribution and two-sample equal variance) in SF vs. AR or CGI categories.

**Figure 5:**
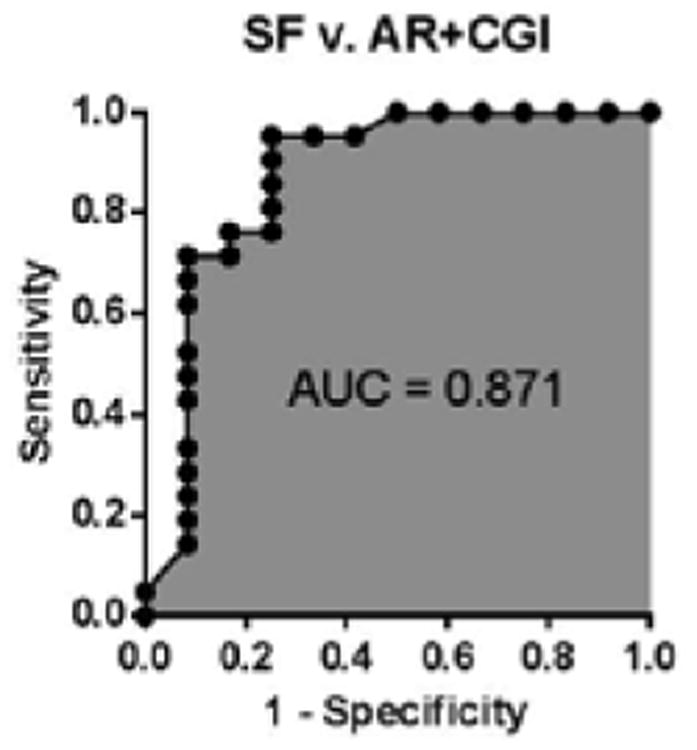
ROC curve for PARP1 discriminating stable function versus acute rejection and chronic graft injury.

The reverse capture arrays with the PARP1 antibody, which was used on Clontech arrays, showed a similar increase in SF compared to AR-CGI and even NK (Figure 4A). Although the AR-CGI-marker PARP1 was identified in a separate analysis as a top CGI-, but not AR-marker, its 2-fold upregulation Profile signified the stabilization of kidney graft function, measured by Protein/Creatinine Ratio (PCR), in a patient having AR episode (Figure 4B). As expected, PARP1 expression showed the opposite, compared to PCR, trend in all patients with the graft rejection/injury (Figure 4C), and the PARP1 upregulation was confirmed in SF vs. AR and CGI (p<0.02 and 008, respectively). In addition, the ROC curves for SF vs. AR and CGI indicate an AUC value of 0.87 suggesting a good discrimination between stable function and rejection states after kidney transplantation (Figure 5). Thus, reverse capture arrays qualified the biomarker role for serum PARP1, first identified by Clontech Ab arrays, and, thereby justified the use of two complementary proteomics platforms in the identification of serum biomarkers in kidney transplantation.

## Discussion

In this pilot study on a limited cohort of patients, a serum proteomics platform empowered by the concurrent GenePattern and IPA analyses enabled the identification of plausible graft rejection biomarkers. Hence, this approach is expected to overcome the multifactorial etiology of allograft damage which constrains the linkage of AR- and ChR/CGI-constituents to the same biomarkers.

**Figure 6:**
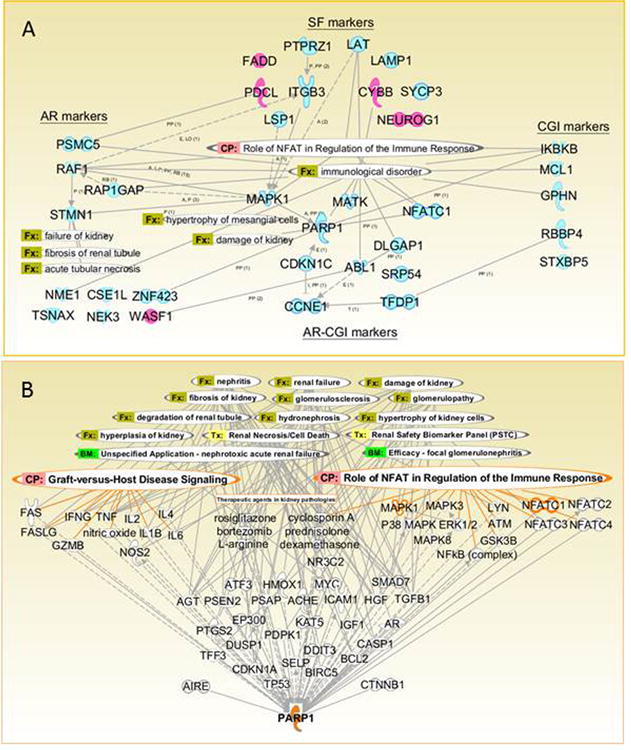
PARP1 and other markers in immune response related signaling and kidney functioning (Ingenuity Pathway Analysis, IPA) Panel A. Along with GenePattern-based identification of candidate markers for stable function (SF) and rejection/injury (AR, CGI, and common AR-CGI), we used IPA for data mining on their interrelations and connections to the kidney-specific functions and the allograft-related signaling. Abbreviations for overlays: BM: biomarker; CP–canonical pathway, Fx–function, and Tx–toxicology list. Abbreviations for interactions (Panel A): A–activation; E–expression; I–inhibition; L–localization; RB–regulation of binding; P–phosphorylation; PP–protein-protein interactions; and T–transcription. Panel A designates possible interactions and their up-(pink) or down- (blue) regulation in corresponding marker category. Panel B shows possible interactions between the major identified marker PARP1 and its multiple interacting partners that can be related to the graft pathophysiology (PARP1, MAPK1 and NFATC1 highlighted). Both networks (Panels A and B) are created using Path Designer, IPA and are shown with the Role of NFAT in Regulation of the Immune Response Signaling and other kidney graft-related overlays.

In the concurrent data mining, IPA substantiated the GenePattern-based selection of putative biomarkers by revealing their interrelations and connections to the kidney-specific and immune response-related functions, which can enable their biomarker roles (Figure 6A). For instance, downregulation of the MAPK1-activating LAT, a major activator of the T-cell mediated signal transduction in immune response, was in line with its SF-marker role. Multiple possible interactions among the markers included direct protein-protein interactions between PARP1 and MAPK1, NFATC1, or ZNF423. Via its interacting partners, the major validated marker PARP1 can be linked to a variety of kidney-related pathologies and drug effects as well as to the Graft-versus-Host Disease signaling that ultimately defines the graft survival (Figure 6B). PARP1 interactions may include beta-catenin, which balances tolerance and immunity [17], and the major AUto-Immune Regulator, AIRE [18]. As a member of the AIRE complex regulating transcriptional machinery, PARP1 can control the AIRE-mediated and thymus-controlled [19] central immunological tolerance in transplantation.

The SF-associated downregulation of *serum* IQGAP1 in our study was in line with its *blood* upregulation in renal allograft rejection [20]. Similarly, the SF-associated downregulation of serum LAMP1 could correspond to its maintained cell surface localization and ‘eat-me’ signaling [6], whereas LAMP1 shedding into the bloodstream could reflect the failing cell survival signature of a malfunctioning graft. In contrast to the SF-associated *serum* PARP1 increase in our study, the nuclear PARP1 increase in kidneys transplant *tissues* has been associated previously with a poorer outcome [21,23]. In addition, the Parp1 gene ablation in homozygous knockout mice decreased kidney damage after ischemic renal injury [23]. To reconcile these seemingly controversial data, it is plausible to suggest that the conundrum of *serum/blood* versus *tissue* Profile in general may be crucial for the determination of biomarker's detrimental or protective role.

The phagocytic Nox2, which modulates fibrogenesis in kidney grafts via the TGFB-associated Smad2 activation [24], was upregulated in SF. About one third of peripheral blood genes that differentiate rejection from stable function is regulated by TGFB [9], which is implicated in epithelial-to-mesenchymal transition, EMT, an integral part of chronic allograft nephropathy (CAN), podocyte dysfunction, proteinuria, and glomerulosclerosis [25,26]. Additionally, the downregulated SF-marker PTPRZ1 tyrosine phosphatase appeared in line with the absence of EMT in SF-patients, since the EMT involves phosphorylation of PTPRZ-substrates and can be initiated by the PTPRZ1-ligand pleiotropin [27].

In our study, unfavorable prognosis in AR-CGI was associated with the MAPK1 and NFATC1 decrease, while SF was characterized by the significant decrease of LAT and ITGB3, and a similar trend of calcineurin and CD3-zeta. The major cause of late graft loss CAN is considered a cumulative lesion involving nephrotoxicity of CNI [25]. Cyclosporin A promotes cell viability via the MAPK3/MAPK1-mediated Ca2+/calcineurin/NFAT signaling [28]. The inhibition of MAPK-kinases can have multifaceted graft-associated effects [29,30], while the interruption of calcineurin inhibition can benefit global graft survival, while increasing the AR risk [31]. Calcineurin downregulates the TCR/CD3-induced expression of LAT, and CNI potentiate the LAT, but block TCR-activation [32]. Vascular CNI toxicity was associated with the arteriolar mural platelet CD61/ITGB3 deposition [33]. This serum Profile, along with the previously reported urine ITGB3 decrease in SF [34] may be useful for defining a patient's eligibility for the calcineurin weaning.

In summary, this study provided the methodology and training sets, which can guarantee a success in larger cohorts of kidney transplant patients with various graft-associated pathologies and treatment modalities. While biomarker/modifier roles of identified candidates need to be elucidated, the AR-CGI markers, such as PARP1, represent a beneficial prospect of non-invasive biomarkers predicating the kidney graft failure regardless of the rejection/injury causes.

## Figures and Tables

**Table 1:** Candidate gender-independent SF-markers for Stable Function in kidney transplant patients Microarray analysis using protein Ab arrays (Clontech-500) with pooled serum samples from kidney transplantation patients and controls was performed, as described in Material & Methods. Candidate SF-markers were identified in SF vs. Rest comparison (4-classes: NK, SF, AR, and CGI) using Comparative Marker Selection (Gene Pattern). Final selection was made using rank, score, feature P, and fold change.

Rank	Upregulated in	Feature	Description	Score	Feature P	Fold Change	SF Mean	SF Std	Rest Mean	Rest Std
1	Rest	P33241	lymphocyte-specific protein 1	-4.143	0.002	1.389	1.274	0.089	1.770	0.385
2	SF	P04839	cytochrome b-245, beta polypeptide (chronic granulomatous disease)	3.983	0.020	1.358	0.630	0.056	0.464	0.108
3	Rest	O76043	protein tyrosine phosphatase, receptor-type, Z polypeptide 1	-3.439	0.002	1.549	1.045	0.157	1.619	0.510
4	Rest	P05106	integrin, beta 3 (platelet glycoprotein IIIa, antigen CD61)	-3.399	0.008	1.284	1.702	0.172	2.185	0.393
5	SF	Q13371	phosducin-like	3.367	0.052	1.323	0.334	0.015	0.252	0.080
6	SF	Q13158	Fas (TNFRSF6)-associated via death domain	3.328	0.018	1.486	1.296	0.236	0.872	0.166
7	Rest	P11279	lysosomal-associated membrane protein 1	-3.293	0.026	1.497	1.036	0.163	1.551	0.463
8	Rest	P70281	synaptonemal complex protein 3	-3.232	0.040	1.288	1.342	0.115	1.729	0.364
9	Rest	O43561	linker for activation of T cells	-3.217	0.036	1.348	1.268	0.165	1.709	0.379
10	SF	Q92886	neurogenin 3	3.140	0.032	1.684	0.276	0.063	0.164	0.059

**Table 2:** Candidate gender-independent AR-markers for Acute Rejection in kidney transplant patients Microarray analysis using protein Ab arrays (Clontech-500) with pooled serum samples from kidney transplantation patients and controls was performed, as described in Material and Methods. Candidate AR-markers were identified in AR vs. Rest comparison (4-classes: NK, SF, AR, and CGI) using Comparative MarkerS election (Gene Pattern). Final selection was made using rank, score, feature P, and fold change.

Rank	Upregulated in	Feature	Description	Score	Feature P	Fold Change	AR Mean	AR Std	Rest Mean	Rest Std
1	Rest	P47736	RAP1, GTPase activating protein 1	-3.733	0.010	1.858	0.278	0.068	0.516	0.187
3	AR	Q92558	WAS protein family, member 1	3.259	0.012	1.638	0.101	0.021	0.062	0.021
4	Rest	P04049	v-raf-1 murine leukemia viral oncogene homolog 1	-3.233	0.022	1.475	0.911	0.208	1.343	0.292
6	Rest	P15531	non-metastatic cells 1, protein (NM23A) expressed in	-2.981	0.048	1.423	0.613	0.106	0.872	0.239
8	Rest	Q99598	translin-associated factor X	-2.819	0.026	1.934	0.038	0.018	0.074	0.031
9	Rest	O08961	OLF-1/EBF associated zinc fnger gene	-2.738	0.012	1.335	0.153	0.032	0.205	0.034
10	Rest	P51956	NIMA (never in mitosis gene a)-related kinase 3	-2.734	0.028	1.328	0.238	0.049	0.316	0.050
12	Rest	P16949	stathmin 1/oncoprotein 18	-2.635	0.026	1.256	0.618	0.050	0.777	0.189
14	Rest	P47210	proteasome (prosome, macropain) 26S subunit, ATPase, 5	-2.447	0.032	1.645	0.087	0.021	0.144	0.071
15	Rest	P55060	CSE1 chromosome segregation 1-like (yeast)	-2.443	0.024	2.104	0.055	0.034	0.116	0.064

**Table 3:** Candidate gender-independent CGI-markers for Chronic Graft Injury in kidney transplant patients Microarray analysis using protein Ab arrays (Clontech-500) with pooled serum samples from kidney transplantation patients and controls was performed, as described in Material and Methods. Candidate CGI-markers were identified in CGI vs. Rest comparison (4-classes: NK, SF, AR, and CGI) using Comparative Marker Selection (Gene Pattern). Final selection was made using rank, score, feature P, and fold change.

Rank	Upregulated in	Feature	Description	Score	Feature P	Fold Change	CGI Mean	CGI Std	Rest Mean	Rest Std
1	Rest	O14920	inhibitor of kappa light polypeptide gene enhancer in B-cells, kinase beta	-3.623	0.008	0.002	0.012	1.276	1.229	0.090
2	Rest	Q96NG9	syntaxin binding protein 5 (tomosyn)	-3.574	0.006	0.001	0.009	1.267	0.141	0.007
3	Rest	Q14186	transcription factor Dp-1	-3.479	0.028	0.017	0.037	1.759	0.265	0.051
4	Rest	P28482	mitogen-activated protein kinase 1	-3.382	0.006	0.001	0.009	1.908	0.181	0.071
5	Rest	P09874	ADP-ribosyltransferase (NAD+; poly (ADP-ribose) polymerase)	-3.269	0.050	0.036	0.062	2.036	0.232	0.067
6	Rest	Q09028	retinoblastoma binding protein 4	-3.175	0.010	0.004	0.015	1.653	0.101	0.015
8	Rest	O95644	nuclear factor of activated T-cells, cytoplasmic, calcineurin-dependent 1	-2.963	0.010	0.004	0.015	2.105	0.068	0.025
10	Rest	Q9NQX3	gephyrin	-2.933	0.018	0.009	0.025	1.255	0.965	0.072
12	Rest	P42679	megakaryocyte-associated tyrosine kinase	-2.746	0.036	0.024	0.046	1.738	0.171	0.046
13	Rest	Q07820	myeloid cell leukemia sequence 1 (BCL2-related)	-2.513	0.028	0.017	0.037	1.911	0.110	0.038

**Table 4:** Candidate gender-independent common AR-CGI-markers for Acute Rejection and Chronic Graft Injury in kidney transplant patients Microarray analysis using protein Ab arrays (Clontech-500) with pooled serum samples from kidney transplantation patients and controls was performed, as described in Material and Methods. Candidate AR-CGI-markers were identified in AR-CGI vs. Rest comparison (3-classes: NK, SF, AR-CGI) using Comparative Marker Selection (Gene Pattern). Final selection was made using rank, score, feature P, and fold change.

Rank	Upreulated in	Feature	Description	Score	Feature P	FDR (BH)	Fold Change	AR-CGI Mean	AR-CGI Std	Rest Mean	Rest Std
1	Rest	P28482	mitogen-activated protein kinase 1	-3.870	0.004	0.260	1.817	0.216	0.062	0.393	0.113
**2**	**Rest**	**P09874**	**ADP-ribosyltransferase (NAD+; poly (ADP-ribose) polymerase)**	**-3.527**	**0.004**	**0.260**	**2.128**	**0.263**	**0.090**	**0.560**	**0.220**
3	Rest	P13624	signal recognition particle 54kDa	-3.472	0.008	0.272	1.565	0.099	0.027	0.154	0.036
4	Rest	Q14186	transcription factor Dp-1	-3.199	0.004	0.260	1.747	0.302	0.075	0.529	0.185
5	Rest	P49918	cyclin-dependent kinase inhibitor 1C (p57, Kip2)	-3.107	0.002	0.238	1.842	0.088	0.019	0.162	0.065
6	Rest	O14490	dynamin 1-like	-3.084	0.002	0.238	1.957	0.050	0.021	0.099	0.039
10	Rest	P24864	cyclin E1	-2.879	0.008	0.272	1.729	0.429	0.048	0.741	0.303
11	Rest	P42679	megakaryocyte-associated tyrosine kinase	-2.868	0.002	0.238	1.832	0.188	0.045	0.343	0.147
14	Rest	Q13691	v-ablAbelsonmurineleukemia viral oncogenehomolog 1	-2.678	0.006	0.260	1.649	0.095	0.020	0.156	0.062
15	Rest	O95644	nuclear factor of activated T-cells, cytoplasmic, calcineurin-dependent 1	-2.483	0.006	0.260	1.940	0.084	0.028	0.163	0.086

## Abbreviations

M: Male
F: Female
AR: Acute Rejection
CGI: Chronic Graft Injury
SF: Stable Function
NK: Normal Kidneys
CNI: Calcineurin Inhibitors
CAN: Chronic Allograft Nephropathy

## References

[1] Mannon RB, Kirk AD (2006). Beyond histology: novel tools to diagnose allograft dysfunction. Clin J Am Soc Nephrol.

[2] Sis B, Mengel M, Haas M, Colvin RB, Halloran PF (2010). Banff '09 meeting report: antibody mediated graft deterioration and implementation of Banff working groups. Am J Transplant.

[3] Kowalski RJ, Post DR, Mannon RB, Sebastian A, Wright HI (2006). Assessing relative risks of infection and rejection: a meta-analysis using an immune function assay. Transplantation.

[4] Cravedi P, Mannon RB (2009). Noninvasive methods to assess the risk of kidney transplant rejection. Expert Rev Clin Immunol.

[5] Kingsmore SF (2006). Multiplexed protein measurement: technologies and applications of protein and antibody arrays. Nat Rev Drug Discov.

[6] Sato H, Azuma Y, Higai K, Matsumoto K (2009). Altered expression of glycoproteins on the cell surface of Jurkat cells during etoposide-induced apoptosis: shedding and intracellular translocation of glycoproteins. Biochim Biophys Acta.

[7] Srivastava M, Eidelman O, Jozwik C, Paweletz C, Huang W (2006). Serum proteomic signature for cystic fibrosis using an antibody microarray platform. Mol Genet Metab.

[8] Paweletz CP, Charboneau L, Bichsel VE, Simone NL, Chen T (2001). Reverse phase protein microarrays which capture disease progression show activation of pro-survival pathways at the cancer invasion front. Oncogene.

[9] Srivastava M, Eidelman O, Zhang J, Paweletz C, Caohuy H (2004). Digitoxin mimics gene therapy with CFTR and suppresses hypersecretion of IL-8 from cystic fibrosis lung epithelial cells. Proc Natl Acad Sci U S A.

[10] Brouard S, Mansfield E, Braud C, Li L, Giral M (2007). Identification of a peripheral blood transcriptional biomarker panel associated with operational renal allograft tolerance. Proc Natl Acad Sci U S A.

[11] Mas V, Maluf D, Archer K, Yanek K, Mas L (2007). Establishing the molecular pathways involved in chronic allograft nephropathy for testing new noninvasive diagnostic markers. Transplantation.

[12] Kang TH, Lee JH, Bae HC, Noh KH, Kim JH (2006). Enhancement of dendritic cell-based vaccine potency by targeting antigen to endosomal/ lysosomal compartments. Immunol Lett.

[13] Aktas E, Kucuksezer UC, Bilgic S, Erten G, Deniz G (2009). Relationship between CD107a expression and cytotoxic activity. Cell Immunol.

[14] Liu YT, Shang D, Akatsuka S, Ohara H, Dutta KK (2007). Chronic oxidative stress causes amplification and overexpression of ptprz1 protein tyrosine phosphatase to activate beta-catenin pathway. Am J Pathol.

[15] Zahedi K, Revelo MP, Barone S, Wang Z, Tehrani K (2006). Stathmin-deficient mice develop fibrosis and show delayed recovery from ischemic-reperfusion injury. Am J Physiol Renal Physiol.

[16] Zahedi K, Wang Z, Barone S, Tehrani K, Yokota N (2004). Identification of stathmin as a novel marker of cell proliferation in the recovery phase of acute ischemic renal failure. Am J Physiol Cell Physiol.

[17] Mellman I, Clausen BE (2010). Immunology. Beta-catenin balances immunity. Science.

[18] Abramson J, Giraud M, Benoist C, Mathis D (2010). Aire's partners in the molecular control of immunological tolerance. Cell.

[19] Pujol-Borrell R, Herrero-Mata MJ, Palou E, Armengol MP (2009). Immunological senescence and thymic function in transplantation. Transplantation.

[20] Alakulppi N, Seikku P, Jaatinen T, Holmberg C, Laine J (2008). Feasibility of diagnosing subclinical renal allograft rejection in children by whole blood gene expression analysis. Transplantation.

[21] O'Valle F, Benitez MC, Gomez-Morales M, Bravo J, Osuna A (2005). Role of poly (ADP-ribose) polymerase in kidney transplant and its relationship with delayed renal function: multivariate analysis. Transplant Proc.

[22] O'Valle F, Del Moral RG, Benitez Mdel C, Martin-Oliva C, Gomez-Morales M (2009). Poly[ADP-ribose] polymerase-1 expression is related to cold ischemia, acute tubular necrosis, and delayed renal function in kidney transplantation. PLoS One.

[23] Zheng J, Devalaraja-Narashimha K, Singaravelu K, Padanilam BJ (2005). Poly(ADP-ribose) polymerase-1 gene ablation protects mice from ischemic renal injury. Am J Physiol Renal Physiol.

[24] Djamali A, Vidyasagar A, Adulla M, Hullett D, Reese S (2009). Nox-2 is a modulator of fibrogenesis in kidney allografts. Am J Transplant.

[25] Jevnikar AM, Mannon RB (2008). Late kidney allograft loss: what we know about it, and what we can do about it. Clin J Am Soc Nephrol.

[26] Liu Y (2010). New insights into epithelial-mesenchymal transition in kidney fibrosis. J Am Soc Nephrol.

[27] Perez-Pinera P, Alcantara S, Dimitrov T, Vega JA, Deuel TF (2006). Pleiotrophin disrupts calcium-dependent homophilic cell-cell adhesion and initiates an epithelial-mesenchymal transition. Proc Natl Acad Sci U S A.

[28] Du MR, Zhou WH, Dong L, Zhu XY, He YY (2008). Cyclosporin A promotes growth and invasiveness in vitro of human first-trimester trophoblast cells via MAPK3/MAPK1-mediated AP1 and Ca2+/calcineurin/NFAT signaling pathways. Biol Reprod.

[29] Wada T, Azuma H, Furuichi K, Sakai N, Kitagawa K (2006). Reduction in chronic allograft nephropathy by inhibition of p38 mitogen-activated protein kinase. Am J Nephrol.

[30] Chen XL, Chen ZS, Ding Z, Dong C, Guo H (2008). Antisense extracellular signal-regulated kinase-2 gene therapy inhibits platelet-derived growth factor-induced proliferation, migration and transforming growth factor-beta(1) expression in vascular smooth muscle cells and attenuates transplant vasculopathy. Transpl Int.

[31] Danger R, Giral M, Soulillou JP, Brouard S (2008). Rationale and criteria of eligibility for calcineurin inhibitor interruption following kidney transplantation. Curr Opin Organ Transplant.

[32] Peters D, Tsuchida M, Manthei ER, Alam T, Cho CS (2000). Potentiation of CD3-induced expression of the linker for activation of T cells (LAT) by the calcineurin inhibitors cyclosporin A and FK506. Blood.

[33] Meehan SM, Baliga R, Poduval R, Chang A, Kadambi PV (2008). Platelet CD61 expression in vascular calcineurin inhibitor toxicity of renal allografts. Hum Pathol.

[34] Srivastava M, Eidelman O, Torosyan Y, Jozwik C, Mannon RB (2011). Elevated expression levels of ANXA1, integrins beta3 and alpha3, and TNF-alpha contribute to a candidate proteomic signature in urine for kidney allograft rejection. Proteomics Clin Appl.

